# Operational data for the risk management of victim operated explosive devices in humanitarian mine action: A Practitioner's perspective

**DOI:** 10.1016/j.heliyon.2024.e25311

**Published:** 2024-01-30

**Authors:** R. Evans, L. Nelson, T. Temple

**Affiliations:** Cranfield University, Centre for Defence Chemistry, Defence Academy of the United Kingdom, Shrivenham, SN6 7LA, UK

**Keywords:** Mine action, Victim operated improvised explosive devices (VOIEDs), Anti-personnel mines (APM), Anti-vehicle mines (AVM), Booby-traps, Operational data

## Abstract

Since Mine Action's inception at the end of the 1980s, operators have collected limited data on the Victim Operated Explosive Devices (VOEDs) they clear. This includes not only data on the explosive ordnance itself but data on how they were found, where they were found and how they were processed and ultimately destroyed. In a context where detection of mines, boobytraps and certain Victim Operated Improvised Explosive Devices (VOIEDs) is an ongoing humanitarian and military challenge, significantly expanded operational data collection provides an achievable way to facilitate enhanced operational risk management. Risk decisions inherent in the clearance of VOEDs are better if made on the basis of extensive operational data. In the absence of a technological solution to detect and positively discriminate VOEDs from false positive indications, the collection of operational data offers the best prospect for “managing” if not “solving” the problem.

## 1 Introduction

To better understand the threat posed by Victim Operated Explosive Devices (VOEDs), including mines, boobytraps and certain Improvised Explosive Devices (IEDs), it is necessary to collect extensive operational data. Operational data can be defined as technical data describing the devices themselves, the location and environment in which they were found, and the process by which they were found and destroyed. Such data can only be collected by those conducting operations on the ground themselves. At present, operational data collection happens in a limited and often inconsistent way, with little standardisation and limited understanding of the potential value of such data to operators themselves. Whether in a humanitarian demining or security context, there is a need to not only understand the problem better, but also really understand our response to the problem, and this can only happen through expanded operational data collection.

VOEDs present a particular risk management problem for both military and humanitarian clearance operators. Many are concealed on emplacement and reliable detection capabilities that can discriminate metal, plastic or explosive components from false positives have yet to be developed [1]. The International Standards Organisation ISO defines risk as the effect of uncertainty on objectives [2]. The concealed nature of VOED contamination and the lack of technology to reliably detect and discriminate makes the risk they pose highly uncertain. In this context, operational methods should be developed to reduce uncertainty to levels as low as reasonably practicable.

The aim of this research was to develop a Clearance Data Model (CDM) that provides a Common Operational Data Set (CODS) for the clearance of VOEDs. The CDM sits alongside a Survey Data Model (SDM) that provides the equivalent for evidence collected as part of the survey process. Together both form an Operational Data Model designed to improve the conduct of operations. This article will describe the Clearance Data Model, development of which enables enhanced risk management through the collection of a much-expanded data specification. The need for accurate and relevant data in order to conduct risk management is established in a range of sectors [3,4]. Adoption of an expanded data model will enable mine action to manage risk in a truly evidence based manner. The clearance data model specification is split into data categories: device data, process data, location data, environment data, and image data. Between them, these categories contain up to fifty data fields per VOED. The data model also proposes significant changes in how mine action data is collected, with the VOED, rather than an area of land, becoming the main unit of data collection. Previously a few attributes might be recorded for an identified area, now up to fifty will be recorded for an individual VOED. The expanded operational data could also enable a substantive role for Artificial Intelligence (AI) in order to identify patterns and calculate risk more efficiently. The wider range of data points provide more potential for algorithms to identify patterns.

## 2 Method – development of the clearance data model

### 2.1 Selection of data

The development of this new clearance data model for VOEDs is based on practical observation of field operations for over a decade. This highlighted not only the lack of standardised approach towards operational data collection, but also the limited extent of the data collected, and the limited utility of that data. From practical observation a set of basic criteria were identified that provided the guiding principles to develop the model.

The first criterion identified was relevance. Deminers are less likely to expend time to collect data they deem to be irrelevant. Thus, relevance was defined by whether operators use the data, or whether it helps them perform their operational tasks. For example, collecting accurate location data using D-GPS may save time by demonstrating that minimum metal mines were placed and have remained in a consistent pattern which means only the ground where the pattern exists needs to be cleared. This saves physical work for a deminer, since otherwise every square meter could potentially have to be fully excavated to a depth consistent with the known explosive hazard. An area of 100 m^2^ where there is reasonable belief there are minimum metal anti-personnel (AP) mines in minefield pattern and where the clearance depth is 20 cm will entail manual excavation of 20 m³ of the topsoil, while wearing PPE; a physically draining and slow task. If the position of each AP mine is plotted with the accuracy enabled by D-GPS, an evidence basis can be developed to justify deminers excavating only those areas which are known to contain mines. In the same hypothetical scenario also consider the depth of the mine found. If the average depth at which mines are found is recorded, and it is 2 cm, with the deepest found at 5 cm, there could be reasonable grounds on which to reduce the required clearance depth from 20 cm and significantly reduce the level of work necessary to clear the area. Alternatively if items are found at 30 cm, a deeper clearance depth would be required. Such a depth implies more physical labour and slower progress by the clearance staff, but the data can at least justify why.

The second criterion was proportionality. Collection of up to fifty data points concerning a single VOED could be deemed to be excessive and impracticable. Just as there is an overall opportunity cost for all the data not collected over decades in mine action, there is potentially a more modest opportunity cost for overburdening field staff with too much data collection. If the process of data collection unduly impedes the physical process of demining, then it may be deemed ill advised. While some data may be repetitive and easy to pre-input into a form, (e.g., the detector serial number for a given deminer), and some data collection might become automated in future (e.g., the electromagnetic signature of a particular mine as indicated on a detector), data collected from the field will overall represent a burden. The burden must always be worth it. The volume of data to be collected must be deemed reasonable, not just by operations managers but also by field staff. The volume must also be manageable by information management staff working directly in support of operations staff. A good benchmark for how much data is manageable is whether senior operations staff can effectively quality control that volume of data. Data that cannot be checked by individuals with a detailed understanding of how to identify potential errors, is data of a potential lower value over time, or even data that is misleading and therefore represents a false depiction of operational results. Therefore, judging the proportional volume of operational data to collect can in some ways be calculated by the capacity to scrutinise that data to identify likely errors.

The third criterion is that of access. Field staff must have access not only to the data they collect, which their demining organisation should provide them with anyway, but also when considering data provided to a national database, to the equivalent national data. If a demining organisation gives data into the system, they should be able to receive the equivalent national data for comparison. To take a simple example, a demining organisation provides the depth at which all the mines of a given model were found for all their operations for as long as they have been operating. They should then also be able to access the equivalent from the national database, the depth of all the same AP mines nationally.

The fourth criterion is data standardisation. In order for the data collected to be comparable, it must be standardised as much as possible. Valid comparisons on data collected on a like for like basis enable meaningful statistical analysis and, as these new data sets grow, will improve the ability to identify statistical difference. Standardisation requires the development of an operational data taxonomy or lexicon. The range of standardised answers to each data field must be developed as a first step. For example the method of detection field should list all the means of detecting VOEDs available in a given operating environment. Typically this could include specific models of Electro Magnetic induction (EMI) and Dual Sensor detectors. It could also include more prosaic methods such as Full Excavation, Rake, Visual, and Mine Detection Dog. Where this process becomes more difficult is for fields where there can be hundreds, even thousands of potential answers. The most obvious field where this is the case is the device model. For example the CORD database contains over 5300 entries [5]. Listing over 5000 models would not be necessary for a given country but conceivably hundreds of items of explosive ordnance, and tens of mines, are realistic. Such a list would also need to be continually updated as new models are encountered. This is especially the case with IEDs that will often require classification and naming for the first time. For example the VS-500 was a new ISIS landmine commonly found in Iraq. It was named by operational personnel on the ground. Such standardisation would require an agreed explosive ordnance classification taxonomy to be developed, ideally on a national basis, but as a minimum on the level of a given field organisation with as much commonality with other national actors as practicable. The risk of data heterogeneity, limiting the ability to make valid comparisons, is currently real in mine action operations, since there is no means to compel the various operators around the globe to comply with a set of minimum data requirements.

The criteria provide the logic for the clearance data model. What data to collect is a choice, since inevitably there are limits to how much data field operators can practically gather. Not everything it might be desirable to know is practical to collect. Relevance, proportionality, access and standardisation all underpin a clearance data model that has utility for implementing operations, data that currently is not collected in many programmes let alone national databases. For example, in theory any marking on a mine that indicates time and place of manufacture could be recorded and this can be relevant, since even slight differences in metal content can change detection potential. However, recording such details accurately at scale is labour intensive and might be seen as disproportionate by field staff and therefore this detail is not listed in the clearance data model, although it could be collected by individual programmes on a discretionary basis.

### 2.2 Categorisation of data

The data collected about individual VOEDs is split into five categories: 1) Location data; 2) data recording the physical environment the devices were found in; 3) data about the devices themselves; 4) data about how those devices were processed (i.e. how the devices were found, removed and destroyed) and 5) raster and metadata data embedded in electronic image files. The five categories of data have the short-hand titles of “Location Data”, “Environment Data”, “Device Data”, “Process Data”, “Image Data”. This can be viewed as the first level in this proposed operational data taxonomy. Within each of these categories the respective attributes with Custom Defined Fields (CDFs) can be listed, ideally on a global basis, although achieving agreement on a country or programme basis is more likely.

## 3 Results

### 3.1 Location data

The key question in demining is “where are the mines?” Accurate position data for all VOEDs, ideally using D-GPS, is a basic information requirement. Currently the position of individual VOEDs is not consistently recorded. If the location for each VOED found and disposed of over the last three decades had been recorded, even if just using relatively inaccurate handheld GPS, a powerful dataset would exist that could be used to compare with other geospatial data. For example, the mines and IEDs on routes present a particular problem, since they are rarely emplaced with such density as to justify 100 % clearance of the entire route, even if resources were available, which invariably they are not for areas 100s–1000 s km in length. Location data allows identification of patterns that can better target resources. For example, if a high incidence of devices near culverts can be identified, scarce clearance resources can be targeted to areas around culverts. In such ways collection of operational data enables management of operational risk. For any stretch of road beyond a few hundred meters in length, limited time and resources means there isn't really such a thing as route clearance at scale. However, there can be risk reduction through targeting clearance resources to where the probability of contamination is highest, a probability that can only be calculated if good position data is collected and analysed as a norm.

In a pattern minefield, (a minefield laid out in a deliberate set pattern, typically by military engineers, with the mines spaced every few meters), location data is also essential; not only to identify the pattern but also to identify mines that have moved from the pattern. During any minefield clearance task a fade out distance will be cleared. This is the distance from the mines on the edge of the pattern from which some form of clearance will be conducted as a safety precaution. How is the fade out distance calculated? There is no standardised way, and it is typically a decision based on ‘informed intuition’. However, location data for each mine, especially any mines that have moved from the pattern, provides an evidence base on which a fade distance can be calculated. In the Falkland Islands the location of each mine was strictly recorded using D-GPS, enabling operations managers to show that no mine on the islands had moved more than 40 cm from its previous position [6,7], and those that had moved at all had been laid on rocks rather than in peat soil. From this data it was possible to implement a very limited fade out for full clearance, backed by a form of much quicker Battle Area Clearance (BAC).

While it has been recognised that “mine action is inherently geographic. It relies on identifying the location and spatial extent of explosive hazards,” [8] this logic has not developed to the extent that recording the position of individual devices has become standard. Unfortunately, most reporting remains based on polygons covering large areas [9]. This represents a failure for mine action as location data for individual VOEDs is nothing short of essential, and the failure to collect it consistently over the course of recent decades represents a fundamental missed opportunity. This is not only because many comparisons with other geospatial data sets are enabled by its collection, but also because the predominant risk management model for mine action, land release, is fundamentally geographic in character [4]. Evidence based risk decisions on land and explosive hazards can inevitably be more refined if the exact locations of those explosive hazards are recorded. At present most location data is no more accurate than association with a site or task area that can be hundreds of thousands of meters squared. Routine collection of location data for each VOED device, preferably exact location data by means of D-GPS, but if not by less exact data by means of handheld GPS, should be a norm. VOEDs should be recorded as points not just ambiguously attributed to polygons. While it would involve extra effort on the part of field staff, the savings enabled by more refined clearance of land where hazards actually exist, informed by analysis of location data, would make such effort more than cost effective.

### 3.2 Device data to be recorded

Device Data firstly requires accurate identification of the device model (e.g., PMN), not something which is routinely collected at present, at least not in national databases. The model has to be collected and it should be explicitly forbidden for field staff to only label an item Unexploded Ordnance (UXO), Abandoned Ordnance (AXO) or “mine” since these terms capture insufficient detail to be of real operational value and field staff should in any case be able to identify the VOEDs they find. If an item model cannot be identified, then it should be recorded as unidentified or unknown. Recording as unknown is in itself an indicator of a device not identified by the organisation. This could mean an ordnance recognition training need for the personnel on site, which would also imply a potential safety issue. For VOEDs models that can be identified, indeed for all explosive ordnance found in the field, the model will give the respective category and sub-category of item. For example if a PMN model was recorded, the category of “Mine, booby trap, or other device” and sub-category of “Anti-Personnel (AP)” would automatically be filled, either on an electronic form or in the database itself*.* For this to be achieved a standardised list of all known VOEDs devices should be established. In this way identification of the model, and how it is inputted, can be uniform, and the resulting data will be comparable.

One issue that currently prevents consistent data recording of VOED models found in the field is the lack of an agreed categorisation system for ordnance. Even within NATO, countries might categorise ordnance differently. For example, sometimes the United States military categorises mortar rounds as a distinct ordnance category [10], and at other times mortar rounds are counted as projectiles [11]. Sometimes in British publications explosive submunitions are referred to as grenades, or contrary to the Convention on Cluster Munitions (CCM), incorrectly as bomblets when this is a specific term for submunitions carried by a dispenser that remains affixed to an aircraft [12]. How landmines are categorised is also not agreed. For example, a POMZ-2M fragmentation mine might be termed a “stake mine” [13] or an “omni-directional mine” [14]. The new International Mine Action Standards (IMAS) classification, while not only containing errors, does not allow for any such detailed classification of a mine like the POMZ-2M, designating it simply an AP-mine [9]. The same is true for all roles of AP Mine. A PMN can currently only be classified as an AP mine, not a blast AP mine. An M − 18 claymore mine can only be classified as an AP mine, not a directional AP fragmentation mine. The same is true of other ordnance sub-categories. A Type-63 Chinese rocket can only be categorised as a rocket, rather than a rocket with a high explosive warhead designed to produce blast and fragmentation. Alternatively a Type-69 Chinese rocket with a shaped charge cannot be categorised as that, only as a rocket. This currently limits how databases can be used to analyse explosive risk.

The lack of an agreed ordnance classification system is one aspect, another is an agreed list of conventional explosive ordnance. Such a list would allow standardised entry of model names into databases, and for drop down lists for electronic forms. A list of VOEDs, such as mines, would be a priority for a standardised list, but given that every item of explosive ordnance can be co-opted into a VOED, whether it is a projectile providing the main charge for an IED, or a grenade forming the basis for a simple booby trap, a standardised list for all positively identified explosive ordnance is also a necessity. There are databases that could provide a starting point for such a list such a CORD [5].

Other elements of device data that should be recorded include the condition of the item by simple binary yes/no/don't know questions. For example, the relevant questions might be damaged, yes/no/don't know, functional yes/no/don't know, alongside signs of weathering, yes/no/don't know. There is a long-standing, and perhaps unique precedent of recording the condition of devices at the point of clearance. Up to December 31, 1945 in Holland, 1,079,857 mines were removed by German soldiers within an ad hoc clearance unit of POWs titled the Draeger Brigade [15]. For about half of the mines lifted (481,329 mines) deminers recorded binary yes/no descriptors to the following questions: “Mines which would NOT have detonated?” and “Mines partially or totally defective?” Data was disaggregated by mine model groups, e.g. Tellermine 35, 42 and 43 [15,16]. How accurate this data might be is open to question, since inevitably it represents a series of possibly rapid assessments by a workforce compelled to conduct dangerous work. There is no evidence the answers to these condition questions was based on testing of components, for example igniters. However, that does not the mean the data is useless. Even if accepted as indicative, the data can still have value. For example, the highest percentages of defective or damaged mines were wooden Schuminen (20.6 %) and Holzenminen (18.4 %), rather than metal cased mines [15]. It might be assumed that the higher “Would Not Have Detonated” percentages would refer to the wooden case mines. The data collected confirms this to be the case, with Schuminen (14.4 %) and Holzenminen (9.6 %) assessed to be not functional [15]. This data collection allows conclusions based on evidence rather than just assumption and anecdote, enabling operational risk management.

The value of these questions can be limited since they depend on the subjective judgement of the form filler, typically the site supervisor. However, with training and guidance, and some clear criteria for the answers, a degree of consistency can be achieved in the collection of condition data. Indeed, Standard Operating Procedures (SOPs) should require that the same individual on a given demining site should fill in forms concerning items found, to increase consistency between entries. Damage and weathering can be assessed visually by someone with a degree of training, but functionality could be harder to discern. For example, in the Falklands assessments of the functionality of detonators extracted from fifty P4B mines removed from four locations on the islands, including a mines dump, found that only 16 (mostly from the mines dump) produced any explosive effect [17]. The testing required for this was specialised and not repeatable at scale across all the clearance sites in mine action. Therefore, a limited visual assessment of functionality, based on a simple yes/no/don't know question is still useful, albeit the limitations of the resulting dataset should be recognised.

The Draeger Brigade also recorded one other significant detail that could be replicated today: boobytrapped yes/no. Amongst allied soldiers there was a common belief that every mine was boobytrapped, typically with some form of pressure release or anti-lift pull device. Stockminen were found to be boobytrapped the most (40.67 %), possibly due to the ease with which a grenade could be employed on the buried portion of the wooden stake. For very common anti-vehicle (AV) mines such as Tellerminen, which had auxiliary fuze wells for the purpose of employing a pull anti-lift device, only 0.87 % were boobytrapped [16]. Whether an item is booby-trapped, or has some form of anti-handling, could be reasonably recorded with a binary yes/no question and should be included as a necessary piece of device data. In any case recording of such data can be seen as a basic form of risk management worth the time expended.

Other details about the devices themselves include the orientation of a mine at the point at which it was found. This could be deemed relevant since approximately 45 % of demining accidents occur at the point of excavation [18], i.e. when the excavation implement makes contact with the fuzing of the device or the soil around the device so that the fuzing is initiated. Arguably if a pressure fuze is orientated away from the horizontal it is more prone to initiation during excavation. On balance while it could be useful to collect this data it is probable any input, in degrees rotated from the horizontal, could be subject to inaccurate data collection. It could also be difficult to collect this data if the means of finding the item was a rake. However, should a given operating environment experience a number of excavation accidents, consideration could be given to collecting this data since it could help provide evidence as to the nature of the explosive hazard, evidence that probably won't be available in any blast scene analysis as part of a post-accident investigation, since the device will have already initiated and for this reason it is included in the clearance data model.

Categorisation of IEDs present a further challenge. Even in their basic description IEDs may have five components: power source, switch, initiator (detonator) and main charge. While IEDs can, and often will use conventional components, they will also make frequent use of improvised components. If data inputs for the five component categories could be agreed, it would greatly benefit how we capture the nature of improvised explosive threat in databases. A United Nations IED lexicon exists [19], which is a direct copy of the Defence Intelligence Agency and JIEDDO Weapons Technical Intelligence and Improvised Explosive Device Lexicon [20]. It provides a basis, although it is not followed in the IMAS [9], and contains some errors [19,20]. There are of course many other pertinent details that could be captured for an IED, including forensic exploitation that is the provenance of security forces rather than humanitarian mine action.

How much device data to collect about improvised landmines or other VOEDs is difficult to judge. In some instances, a similar amount of data as is collected for an equivalent landmine could be suitable. In Iraq and Syria, the locally named VS-500 can be categorised as an IED, an improvised landmine and both an AP and AV mine. It has a simple mechanical fuze and a significant main charge (8 kg), albeit typically one of aluminized ammonium nitrate. However other devices, that also have fuzing or a switch utilising pressure, often a 1 m pressure switch using copper wire, or a crush wire switch, will have more separate parts that would require more device data fields to accurately record. The data model could also allow for recording of multiple initiation switches if necessary. As a minimum for any device that could be designated an IED, agreed descriptors for the five basic components should be recorded in national databases. Where such descriptors are less relevant, e.g., power source for a mechanical fuze, rather than an electronic circuit making use of a battery, relevant information, e.g., mechanical, can still be recorded. Such information is sufficient for civilian mine action purposes. Security related clearance operations would possibly require significantly more IED device data.

### 3.3 Identifying process data

Process data should detail how mines, or equivalent IEDs and booby traps, were found and removed. It might be assumed that how this process is achieved is known, but invariably understanding is anecdotal. It is also important to note that process data is not exclusive of device data. Analysis in conjunction with device data is essential for risk management.

All process data should be attributed to an individual VOED device, as such an AP mine. The unit of process data could be fairly deemed to be the clearance site, albeit for spot tasks, the clearance site may well be for one VOED device only. Recently for the first-time site context has been recommended, but not required as standard [20]. However it is better if the unit is the immediate area around the device and is associated with the device itself. Inevitably this will involve repetition of information in data forms for a scenario where a number of VOEDs are removed from the same area. For example, if that area was originally desert with hard baked soil and a former battlefield with significant metal contamination, those details might be repeated for each device. Alternatively collecting by area units captures inaccuracies, since for a given site conditions can vary significantly. Therefore, the only way to capture the range of conditions, rather than an approximation of the conditions that dominate across an area of potentially many hectares, is to record the data associated with each VOED device. Whilst this may seem repetitive, replication can be automated in electronic forms. It is also advantageous if there is consistency in data collection between EOD spot tasks and area clearance where practicable. Standardising the association of process data with individual device units is the logical way to achieve this.

The first item of process data that should be collected, and perhaps the most important, is data on how an item was found. The choices here might include visual, full excavation (hand), full excavation (rake), electro-magnetic induction handheld sensor, dual sensor incorporating ground penetrating radar, and Mine Detection Dog (MDD). Other options in time might include visual by means of UAV and infrared identification, again by means of UAV. One unfortunate category of how the item was found could be through “detonation” that could also indicate an incident, and possibly an accident.

Associated with the found heading should be a simple identification of the individual who positively identified the mine. Given the potential constraints of data protection legislation, it is possible identification of individuals by an assigned ID number is preferable, but in any case, the individual should be recorded. From Vietnam to Afghanistan, visual identification of VOEDs has been a consistent theme [[21], [22], [23]]. Some individuals are more effective than others when it comes to visually identifying the presence of VOEDs [24,25]. This even includes visual identification by optical devices on UAVs [26]. It is important those individuals can be identified through data. Every means of detection, regardless of tool or sensor, still requires a human decision to adjudicate the information presented. Even if a MDD finds a VOED, their identity, and very importantly the identity of their handler, should be recorded in relation to that individual VOED.

If the individual used a sensor to detect a VOED, the individual identity of that sensor should be recorded. For a handheld EMI or dual sensor detector this will mean recording the serial number. If every VOED found by a handheld sensor could be linked to the exact identity of that sensor and possibly even its settings at the time of detection, the risk management benefits could be very significant. Consider a scenario of two detectors being used on a site. After a given period of time, one detector has found ten VOEDs, and another has found zero. There might be reasons for this disparity, including the conduct of the operator using the detector, and where each operator has been assigned to search. One area might contain mines, another not. But the key is the collection of data allows the disparity to be identified and an explanation to be sought. If the detector has a fault not picked up at the site test pit, that potential fault can be identifiable from the data, but only if the data is collected and analysed. Detectors often represent a substantial outlay for a given demining programme, the capital expenditure on which often figuring significantly in start-up costs. If data were collected on what detectors find what mines in what circumstances, such decisions can be made with hard evidence. Recording of such a simple data point also enables monitoring of equipment performance over time. For example, hypothetically comparison with depth data might identify a scenario such as a particular serial number progressively identifying mines with comparable signature at shallower depths over time. Alternatively it could show no depreciation in performance. The overarching point remains that it is the collection of data that furnishes an answer, or at least points towards further relevant questions, and enables better operational risk management.

Time is key process data, chiefly the time at which items were detected, and the time at which they were destroyed. Are deminers more alert at a given time in given environmental conditions? In a hot climate will deminers be alert during cooler conditions in early morning? If the time of day when items are detected was known, we would have data on which to assess this question rather than just anecdotal feedback. It could be that such data would reveal no particular pattern concerning time of item discovery, but even in such circumstances knowing there is no pattern enables operational staff to reduce uncertainty in their decision making. The time between discovery and destruction of a VOED can also be deemed to represent potential risk. On many demining sites in areas where deminers don't have the explosive means to destroy items themselves, and are not permitted to burn ordnance, items can be left in place for many days until the mandated government agencies come to conduct disposal. Leaving items in place, especially overnight when sites are not staffed, represents a safety and security risk. Recording the time of finding and destruction enables identification and measurement of that risk.

The process associated with searching or clearing should also be recorded, for example whether vegetation had to be removed prior to or during clearance, and how it was removed. It could have been removed by burning or mechanically by plant with a mower head or flail or removed manually by a deminer in their lane. The deminer may have used a strimmer or might have used a slower method using garden cutting implements. Sometimes it can be a combination with initial removal of main vegetation mechanically, and final removal of lower vegetation by hand.

Data on whether the ground was mechanically processed prior to items being found and removed should be recorded. In the past, areas of AP mine contamination might be processed with a flail or tiller many times, the thinking being that such machines would destroy the fuzing mechanism of any items in a given area while loosening hard soil. This would, so it was believed, make the subsequent manual clearance easier. However, such approaches are no longer favoured. Items, including fuzing systems, can remain functional after the ground has been processed by a tiller or flail. Also, especially for items such as minimum metal mines or low metal content IEDs, if items are placed in some sort of pattern, the pattern is the main indication as to the position of mines that EMI detectors might not be able to discriminate from other metal contamination in the soil. Machines destroy the pattern and in doing so destroy key evidence indicating the location of items. Therefore, machines tend to process up to an area suspected on containing mines and not process over it. If mechanical assets were used to access the immediate area of a VOED, this should also be recorded under a means of access heading.

How and where devices were disposed of is important information. Were items moved prior to destruction, and if so, was some form of neutralisation and disarming conducted first? It might be believed that most VOEDs are destroyed in situ where they are found. Clearance personnel will usually not do this unless there is a compelling safety reason, such as a suspected anti-handling device that precludes moving the item or requires neutralisation itself, a process that might entail unnecessary risk. However destruction in situ by high or low order techniques risks spreading further fragments (possibly metallic) around the immediate area. Each of these could be a signal for an EMI detector that will require investigation, slowing subsequent clearance. Therefore if VOEDs can be moved they will be. Sometimes items that some might not move, are moved; for example, AP mines that incorporate a cocked striker fuzing mechanism that can potentially become more dangerous over time as the item weathers and it holding devices deteriorate. One basic way of reviewing risk at a national level could be to list all mine models that include a cocked striker mechanism, such as a PMN, and then compare with the incidence of moving items for destruction at another location. If such a practice does entail risk, as anecdotally is believed to be the case by some, the data will provide actual evidence as to whether this is really the case. Sometimes items, especially AV-mines or IEDs that might be booby trapped, are “pulled” remotely. This should be recorded with a simple Yes/No question. A destroyed in situ Yes/No question should also be included. If only components of the item are moved prior to destruction, such as might be the case with a pressure plate ID, questions in the data collection form should also be able to capture this scenario. Neutralisation and disarming are often collectively referred to as a Render Safe Procedure (RSP) [27,28]. A neutralisation procedure can entail significant risk and would typically be done by the senior EOD technician on site. In the same way that the time of finding and the time of destruction should be recorded the time of neutralisation should also be captured in any operational dataset. In addition, the identity of the individual conducting neutralisation or disarming should be recorded.

How items are moved should also be recorded. It is one thing for the senior EOD qualified individual on site to move an item in PPE on the basis of one person one risk, it is another for individual deminers to do so as moving items is a particularly high risk activity. As with much risk management understanding exactly what actions are being conducted enables pertinent risk management. Movement of certain items by vehicle presents particular risk if those items have not been subject to an RSP as required. Possible answers to a moved yes or no question could be no, yes (manually) or yes (vehicle). The recording of these activities, alongside just the model identity of a conventional item, enables not only oversight of risk being followed in the field, but also compliance with accredited SOPs, the main instrument of risk management in mine action.

### 3.4 Data to capture the environmental context

The environment where a VOED is found should be recorded, at least in some detail, as it was prior to the beginning of clearance. Where clearance of VOEDs takes place can be as relevant as the actual VOEDs being cleared. The environment within which such devices are located has a fundamental impact on how easy it is to find and clear those devices. At present datasets tend not to record environmental data, at least not in relation to the specific device. No Information Management System for Mine Action (IMSMA) database records environmental data. There is a precedent of sorts. In 1945 general observations of mine ageing in Holland were recorded. Land was categorised into three general descriptions: “Sandy and Dry Earth, Low Lying Meadows and Polderland which has been inundated since the mines were laid.” The ageing effect of such conditions on metal and wooden cased mines was noted, with wooden mines more likely to age to the point of non-functioning in a few years [15]. The capture of environmental conditions was general, and not in relation to individual items. However, this was 1945, and data was collected using simple paper forms. Collecting more environmental data today, using handheld devices, in relation to individual devices rather than areas, would be more practical.

Many areas that have been mined, or thought to be mined, have been avoided by humans for prolonged periods, sometimes many decades. In certain environments this will lead to significant vegetation growth within the mined area. Such conditions should be recorded. Options could include, primary jungle, secondary jungle, grassland, desert etc, with clear definitions to improve standardisation of answers. Another environmental factor that should be recorded is soil, not least soil hardness. This can be done recording a process such as “watering required?”, yes/no, since this implicitly details whether the hardness of the ground justified the expenditure of time and resources to alleviate this. In countries like Sri Lanka, even in sandy soil, water bowsers are required on demining sites for this purpose.

Other aspects that should be recorded include metal contamination, since this is the factor that anecdotally slows clearance more than any other. How this might be recorded in a useful way is difficult to judge since binary questions don't really suffice when trying to record degrees per contamination. One way might be recording average number of false positive indications per meter squared where the VOED was found. This is not as straightforward as it might seem. One of the only instances when every item of metal contamination was recorded during the course of clearance was a trial the Valon Minehound dual sensor from September 2012 to December 2013. Two detectors checked 197,044 signals, 99 % of which were clutter. For each mine found by the Minehound 32,841 metal signals were investigated [29]. While trials such as this indicate that the cost in time required to record the number of false positives for each deminer is not prohibitive, whether that cost would be accepted in a competitive mine action sector, and not just during a subsidised trial, is unclear. Time spent counting and reporting false signals onto forms, even electronic forms, is arguably time not spent for deminers progressing a clearance lane. However it is possible that a simple “clutter count” could be done without too much disruption, especially if done by deminers at the end of the working day. Items of clutter could be counted, and then divided by the meter squared cleared to get a number/m^2^. Were that cost to be deemed worthwhile it could provide an extremely useful context indication and allow a much greater understanding of how practicable it is to search an area using metal detector technology. It could also be deemed essential if recording of indication signatures, including false positives, for a range of sensors, is required for the development of sensor technology.

Topography of where the device was found can also be valuable, with the inclination of the ground where the device was found recorded. As with much data capture the field staff filling in the forms will need not only training to enable them to do so accurately, but also a quality management system to monitor information gathered from the field. If location data is recorded accurately it is possible topography can be ascertained from location data, rather than a physical measurement by a deminer in the field.

Recently recording of basic environmental information on a site level has been recommended, but not mandated, in a Technical Note for Mine Action. This is an advisory document that explicitly does not constitute a standard and therefore does not amount to a requirement [30]. Questions that record environmental conditions for a site include estimated slope angle, hard soil Y/N, significant coverage prior to processing Y/N, significant rubble Y/N and so on [30]. These questions are perhaps a crude means of capturing context in an area, and as already described don't allow for variations on site. However no context capture for operational datasets had been tried before, certainly not in national IMSMA databases; so the intent was to introduce the mine action community to the concept in a limited way. Ideally the next step would be to sharpen the question and relate the data to each item found, even if it risks repetition between entries for items found on the same site and potentially subject to the same environment. The extra work is worthwhile in order to record variation within a site. For example, perhaps some mines might be found on a slope with a 30° angle, and other at a 10° angle. Relating the specific environment to the device enables this variation to be recorded, and its implications analysed. Using the VOED device as the key unit of data is the logical next step in order to improve our understanding of the nature of the VOEDs encountered in the field, and improve the risk management of how those items are found and disposed of.

### 3.5 Collecting photographic data

Potentially an image could be taken of every VOED found in the field at the point when it has been excavated sufficiently in order to positively identify. Capturing such data would involve a significant expansion in the data storage capacity required, in a context of already vastly expanded number of device, locations, process and environment data fields. However, the process of taking the image is not overly costly in time, and the resulting image bank can add a powerful new dimension to any operational dataset. For operations managers conducting quality control of their operational data, it provides a visible means of checking the written and numerical data they receive. For example, an operations manager that examines a data row detailing a PMN mine found damaged, assessed due to burning, would address an inconsistency if the corresponding image showed a similar looking Gyata-64 mine in good condition instead. From this the manager could focus on other data from the same source to quality control, and also be aware of the need to re-train or potentially discipline. This would all be in a working context where accurate data collection becomes fundamental to the role of operational field staff and their job performance would be assessed accordingly.

Images taken of items at the point of maximum excavation could also enable a powerful means of remote quality assurance. According to such data as exists, excavation is one of the most dangerous activities for both demining and for clearance of VOIEDs [31], and visual records of this work can enable managers to check on each excavation procedure. If the item was moved, a second image can be taken. This image would also provide indicators as to whether it was appropriate to move that item. For example, if an item with a cocked striker is moved, and the post move image does not show the booster and detonator assembly as removed, this is a clear indicator to the operations manager that immediate remedial training is required. Normally movement of an armed PMN, especially one subject to weathering, and thus where the cylinder spring might not support the cylinder stop holding device, is strictly forbidden.

Images with a date time stamp, and an accompanying location would also provide an important check on fraudulent field reporting. Each image could have a geotag such as Exchangeable Image File Format (EXIF). This can be compared with and act as a quality check on more accurate D-GPS data if available. The timestamp also adds another barrier to operational data fraud since it would need to be consistent with the time between the discovering of the VOED and its destruction. Such cross checking can be automated. The result is renewed confidence throughout the chain concerning the product, VOEDs cleared and destroyed, that is being funded. From operations managers at various levels within demining organisations, to national authorities, to donors, the capture of all extra relevant data enables enhanced transparency. The capture of even one standard image per VOED, taken to a standard format, has advantages beyond just professional interest for those supervising field clearance. Imagery also enables greater connection between those funding clearance and what they are funding. Individuals could fund individual mine clearance with the ability to identify the specific mines their funding cleared, with an individual VOED identification number in the database, each with a line of data and the accompanying image.

Standard image capture does present a potential data storage issue that could have a cost and logistics implication. However, such an issue is not prohibitive and one jpeg file per VOED, captured in a standard reduced format, presents a modest data burden compared with much modern data use. The standard requirement for imagery in operational databases would also only extend to VOEDs and not to other explosive ordnance, unless that ordnance was a relevant component of an IED, booby-trap or supplementary charge for a mine.

### 3.6 Use for incident and accident investigation

One additional benefit of the data collected through the CDM model is the evidence it can provide for accident investigation. To date such data that was collected was mainly concerned with injuries [18,31,32]. For example, as previously discussed, there is some data to suggest that excavation is the most dangerous demining activity and that typical excavation accidents involving AP mines result in traumatic amputation of the excavating hand, the spare hand if not behind the back, and the face, if the visor is not seated in the double collar of the Kevlar apron. The context for such accidents can be provided by the recording of the individuals conducting excavations relative to device model for the first time. Other additional data that could be considered include more process data detailing the time taken to conduct excavation. If the time for each excavation for a specific mine model in each context for each deminer was recorded, and then that deminer had an accident whilst excavating, their history could be key evidence in any root cause analysis.

## 4 Discussion

### 4.1 The cost of collecting operational data

**Fig. 1:**
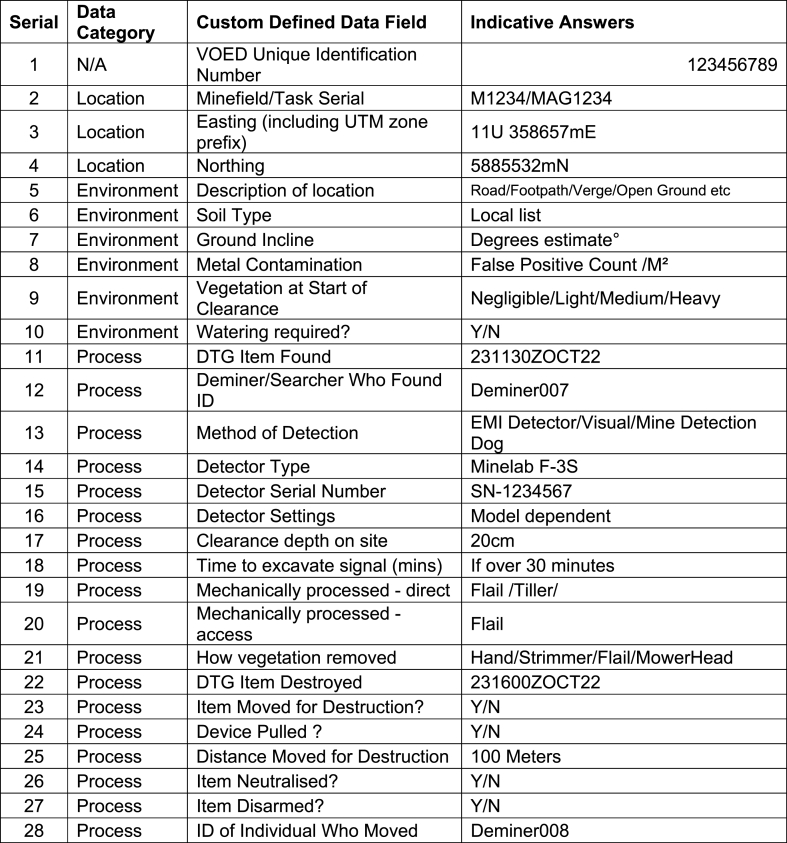

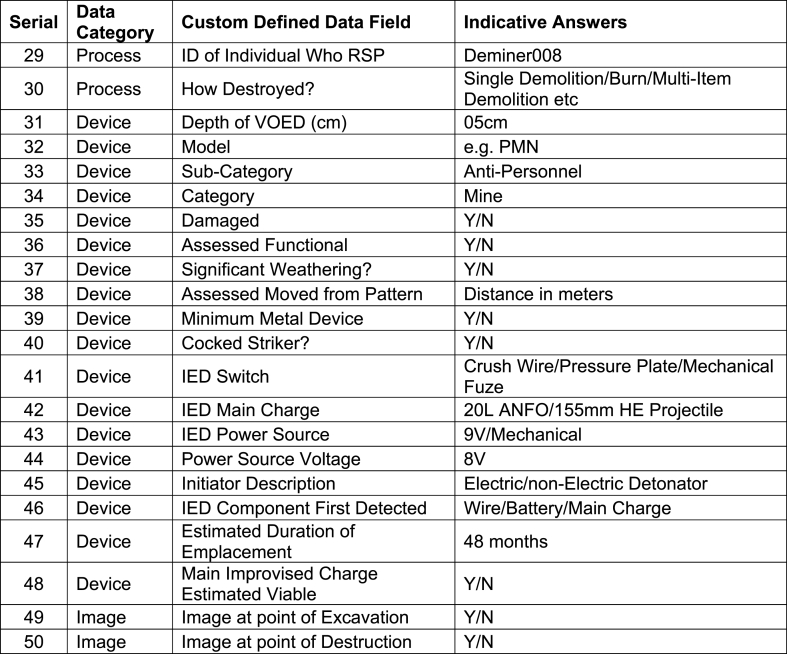
Clearance Data Model for Mine Action

A summary of the proposed data to be captured is presented in Fig. 1 and entails up to fifty data points for one VOED, dependent on if the device is an item of conventional ordnance, such an AP mine, or an IED, which would entail more data points. On sites where it has been known to remove up to 400 AP mines a day, such a Mulihammai in Sri Lanka, that could entail approximately 16000 data entries a day, a significant data entry burden. Nevertheless it is worth the effort to collect this operational data and it is practicable to do so.

On every clearance site there are team leaders and possibly section commanders, dependent on team size, who perform a supervisory role. These individuals invariably have time to record the relevant operational data since they are not conducting the actual physical clearance. Even if the data collection is too much of burden for site supervisors, there other staff such as drivers and medics who have time during the working day to capture the data accurately. For EOD teams, including IEDD teams, the lead operator is backed by a team including a Number 2 who have sufficient time to capture the relevant task data. For all, the imperative to capture the operational data with the maximum accuracy would be emphasised in their respective job descriptions. It would be something on which they would be constantly assessed. The aim would be to develop a culture amongst operations staff where data collection is seen as second in importance only to safety, and a recognition that the two are intimately connected, since operational data enables operational risk management.

A further cost of data collection would be the increased training burden for operations staff. Such training would need to be rigorous, with candidates showing they are able to record data to the necessary standard and in accordance with criteria. Arguably such training need not result in a significant expansion of training time. Nevertheless the capture of relevant data should become integral to EOD, deminer and survey courses in a way that they are not at present. International Mine Action Standards, National Standards and Standard Operating Procedures should all also reflect the imperative of collecting operational data.

### 4.2 The limitations of data

The collection of operational data does not represent any form of full solution to the problem of finding and clearing VOEDs. However, it is what can be done now, and does represent a means to really understand the VOED threat. Without this understanding of the “complexion” of the problem, the efficiency, effectiveness and risk management of efforts to find and clear VOEDs will remain inhibited, in both humanitarian and military contexts.

While limited data collection concerning explosive devices was an important development during the Vietnam conflict [[33], [34], [35], [36]], it is also important to note that Vietnam also showed the limitations of decision making based on data, or at least the limitations of decision making based on erroneous data or misinterpretation of data. What became known as the McNamara Fallacy or qualitative fallacy [37], involving use of data and adherence to metrics to the exclusion of any qualitative context, was coined more towards erroneous military metrics such as body count. One critique of prioritising data states; “the first step is to measure whatever can be easily measured. This is OK as far as it goes. The second step is to disregard that which can't be easily measured or to give it an arbitrary quantitative value. This is artificial and misleading. The third step is to presume that what can't be measured easily really isn't important. This is blindness. The fourth step is to say that what can't be easily measured really doesn't exist. This is suicide [38].” That is not an argument for not spending the time and effort to centralise relevant data collection in operational training, practice and culture. However, it is an argument for care to be taken in how operational data is interpreted, with due acknowledgement of potential limitations. It also underlines the care required in data collection. The moment the process of collecting operational data is not given necessary importance by all involved, the quality of that data can reduce significantly to the point where it does not just become of limited use but also potentially misleads and has a negative effect on risk decision making. Those using operational data must develop a healthy scepticism, and an ability to constructively question and cross check the data on which they may make very significant decisions. Once collected, analysis of it will illuminate operations in a way not possible before, when understanding was overly based on anecdote. If the analysis of data, and the link between real operational decision making is transparent, the extra time taken in collecting data will not necessarily be seen as an impediment to more critical tasks, but a means to work more intelligently and often save time and effort accordingly. Deminers might tire of collecting VOED device depth data, but if it leads to an evidence base that justifies reducing clearance depth, the collection of data can prove ultimately to be justified.

## 5 Conclusion

It would be false to claim that collection of data represents a panacea or silver bullet for finding and clearing VOEDs. There are clear examples of the historical limitations of data, whether in Vietnam [33], or during the counter-IED effort of the last two decades [39]. There are also clear examples of the limitations of data for HMA during the same period [40]. However, in the absence of a technological solution to detect and positively discriminate VOEDs, the collection of better data offers the best prospect for effectively “managing” if not “solving” the problem.

Development of the CDM as part of the ODM represents a novel departure for Humanitarian Mine Action. The expansion of operational data based on the criteria outlined has never been attempted before in the sector. Collection of the location, device, process, environmental data attributes offers the prospect of producing the most detailed set of relevant operational data ever recorded, that enables quantitative risk management based on evidence. Data collected by field staff for better decision making by field staff should become a norm in the sector in a way it is not at present.

A field trial will be necessary to assess the practicalities of vastly expanding the collection of standardised operational data in the field. This should be conducted with trained teams conducting live demining. The key will be to deduce what level of data is realistic to collect, whether the data attributes selected have utility, and whether other data should also be considered for inclusion in the CDM. Other practical aspects such as the best electronic means for data collection in the field, and quality control of data once collected, will also be tested.

The collection of relevant data, by operations staff for operation staff, represents a radical culture shift for those working in HMA, commercial companies and the military. Accurate collection of relevant data should be classified as a key function of all field operations staff, not something to be done grudgingly, and assigned a low priority in comparison to other field tasks. The function of operational data collection should be a crucial facet of operational EOD, searcher and demining training. Only safety should be accorded a higher priority. Operations staff should actively see themselves as data gatherers. They should be assessed not just on the results of their tasks, such as the clearance of an area, or removal of a given VOED, but also on the quality of the data they have collected. With better operational data mine action can know itself and manage its risks better. It can understand not only the problem of VOEDs more deeply but also understand how it can be addressed more effectively.

## CRediT authorship contribution statement

**R. Evans:** Writing – review & editing. **L. Nelson:** Supervision. **T. Temple:** Supervision.

## Declaration of competing interest

The authors declare that they have no known competing financial interests or personal relationships that could have appeared to influence the work reported in this paper.
